# Adipokine Profile Signature in Adolescent Girls with Menstrual Disorders and Hyperandrogenism Differs from That of Regularly Menstruating Girls

**DOI:** 10.3390/jcm14227987

**Published:** 2025-11-11

**Authors:** Elżbieta Foryś, Agnieszka Drosdzol-Cop, Ewa Małecka-Tendera, Aneta Monika Gawlik-Starzyk, Karolina Skrzyńska, Magdalena Olszanecka-Glinianowicz, Agnieszka Zachurzok

**Affiliations:** 1 Department of Pediatrics, Faculty of Medical Sciences in Zabrze, Medical University of Silesia in Katowice, 40-055 Katowice, Poland; azachurzok@sum.edu.pl; 2 Department of Gynecology, Obstetrics and Gynecological Oncology, Faculty of Health Science in Katowice, Medical University of Silesia in Katowice, 40-055 Katowice, Poland; adrosdzol@sum.edu.pl; 3 Department of Pediatrics and Pediatric Endocrinology, Faculty of Medical Sciences in Katowice, Medical University of Silesia in Katowice, 40-055 Katowice, Poland; etendera@sum.edu.pl (E.M.-T.); agawlik@mp.pl (A.M.G.-S.); k.skrzynska@sum.edu.pl (K.S.); 4 Health Promotion and Obesity Management Unit, Department of Pathophysiology, Faculty of Medical Sciences in Katowice, Medical University of Silesia in Katowice, 40-055 Katowice, Poland; molszanecka@sum.edu.pl

**Keywords:** adolescent girls, hyperandrogenism, PCOS, adiponectin, apelin, leptin, omentin, retinol-binding protein 4 (RBP-4), resistin, vaspin, visfatin

## Abstract

**Introduction**: Polycystic ovary syndrome (PCOS) is associated with obesity, numerous metabolic complications, and an increased risk of cardiovascular disease. Adipokines, secreted by adipose tissue, may contribute to the development of these cardiometabolic disturbances. The aim of this study was to investigate the adipokine levels and their relationship with metabolic status in adolescent girls with PCOS. **Patients and Methods**: This cross-sectional study included 66 adolescent girls with PCOS (mean age: 16.5 ± 1.08 years; study group, SG) and 30 regularly menstruating girls (mean age: 16.2 ± 1.37 years; control group, CG) recruited between 2012 and 2017. All participants underwent physical examination, body composition assessment, liver ultrasonography, and biochemical and hormonal evaluations. Fasting venous blood samples were collected to determine the adipokine profile, and the leptin-to-adiponectin ratio (L/A) was calculated. **Results**: Compared with the control group, the PCOS group demonstrated significantly lower adiponectin (*p* = 0.019) and vaspin (*p* = 0.037) concentrations, and higher RBP-4 levels (*p* = 0.048). Positive correlations were observed between adiponectin, apelin, and omentin, while negative correlations were found between leptin and L/A and HDL cholesterol levels in the SG. Omentin showed a negative association, and leptin and L/A a positive association, with triglyceride concentration. In the SG, resistin and visfatin levels were negatively correlated with total cholesterol, and resistin also showed a negative correlation with LDL cholesterol. In patients with PCOS, adverse associations were observed between carbohydrate metabolism parameters and insulin resistance indices, while insulin sensitivity indices correlated positively with adiponectin and omentin concentrations. Visfatin levels in the SG correlated negatively with QUICKI. **Conclusions**: The adipokine profile of adolescent girls with PCOS differs from that of regularly menstruating peers, particularly in adiponectin, RBP-4, and vaspin concentrations. However, the absence of significant correlations between RBP-4 and vaspin and lipid or carbohydrate metabolism parameters suggests that these adipokines are not reliable markers of metabolic disturbances in adolescent girls with PCOS.

## 1. Introduction

Polycystic ovary syndrome (PCOS) is one of the most common endocrinopathies, with an estimated prevalence ranging from 6% to 22% among women of reproductive age [1]. Patients with PCOS are at an increased risk of developing conditions associated with metabolic syndrome. Overweight and obesity, dyslipidaemia, insulin resistance (IR), impaired glucose tolerance, and type 2 diabetes mellitus (T2DM) occur more frequently in this population than in the general population [2]. It is estimated that up to 80% of women with PCOS in the United States and 30–50% in other countries are overweight or obese [3].

The syndrome is frequently diagnosed during adolescence, with its prevalence estimated to be as high as 16.3% based on modified Rotterdam criteria [4]. At the time of diagnosis, up to 48% of girls present with a body mass index (BMI) above the normal range [5].

Adipose tissue is not merely an energy reservoir but also an active endocrine organ that synthesises and secretes numerous bioactive substances, including adipokines. These adipokines contribute to maintaining homeostasis through autocrine, paracrine, and endocrine mechanisms. They regulate appetite and satiety, influence carbohydrate and lipid metabolism and overall energy balance, participate in angiogenesis and vascular remodelling, modulate blood pressure and coagulation processes, and affect the reproductive system [6]. Adipose tissue is a dynamic organ, and its adipokine profile varies depending on its quantity and physiological state. Obesity leads to dysregulated adipokine secretion, which may play a pivotal role in the development of IR, T2DM, and an increased risk of cardiovascular disease.

A growing body of evidence suggests that abnormal adipokine secretion contributes to the pathophysiology and prevalence of metabolic disturbances in adult women with PCOS. Endocrine dysfunction of adipose tissue may represent an important link in the pathogenesis of PCOS, while obesity—particularly visceral obesity—is a significant risk factor for the development of the syndrome in genetically predisposed women. Visceral adipose tissue (VAT), a metabolically active fat depot, secretes numerous adipokines that may substantially influence the development of endocrine and metabolic abnormalities in PCOS [6].

The primary objective of the present study was to evaluate the adipokine profile in adolescent girls with PCOS. A secondary objective was to examine the associations between adipokine concentrations and parameters of carbohydrate and lipid metabolism. In the present study, we hypothesised that the adipokine profile differs between patients with PCOS and adolescent girls with regular menstrual cycles who do not exhibit clinical signs of hyperandrogenism. Furthermore, we assumed that the concentrations of individual adipokines may serve as potential markers of PCOS development or of metabolic abnormalities associated with this syndrome.

## 2. Material and Methods

This cross-sectional study included 66 adolescent girls with PCOS (study group, SG), diagnosed according to the modified criteria proposed by Teede et al. [7] (chronological age: 16.5 ± 1.08 years; gynecological age: 4.2 ± 1.6 years), and 30 regularly menstruating girls without clinical signs of hyperandrogenism (control group, CG) (chronological age: 16.2 ± 1.37 years; gynecological age: 4.3 ± 1.4 years), in whom hormonal disorders were excluded. Diagnostic parameters of PCOS in SG and CG are presented in Table 1. The control group was matched to the study group for both chronological and gynaecological age (Table 2).

Participants were recruited between 2012 and 2017 from patients of the Department of Pediatrics and Pediatric Endocrinology. All participants were of Caucasian ethnicity.

Exclusion criteria included the absence of consent from the patient or their legal guardian; the presence of eating disorders such as anorexia nervosa or bulimia nervosa; thyroid dysfunction (thyroid-stimulating hormone [TSH] concentration < 0.4 mIU/L or >4.2 mIU/L); or adrenal cortex dysfunction, defined as 17-hydroxyprogesterone (17-OHP) concentration < 2 ng/dL. In cases with 17-OHP concentrations between 2.0 and 9.9 ng/dL, a 24 h urinary steroid profile test was performed to exclude congenital adrenal hyperplasia.

Additional exclusion criteria were hyperprolactinemia (prolactin concentration ≥ 721 mIU/L), the use of hormonal therapy within the three months preceding the study that could affect sex hormone levels, and gynaecological age below two years.

The study was conducted according to the Helsinki Declaration and was approved by the local bioethics committee (no. KNW/0022/KB1/95/I/12, no. KNW/0022/KB1/95/II/12/15). Informed written consent was obtained from every patient’s parents/guardians and from every patient.

The following data were analyzed for all patients:

1. Anthropometric measurements: body weight, height, and waist circumference were measured using standard procedures. Body weight was recorded to the nearest 0.1 kg, and height to the nearest 0.1 cm using a Harpenden stadiometer (Holtain Ltd., Crymych, Wales, UK). Body mass index (BMI) and the BMI standard deviation score (BMI z-score) were calculated. The severity of hirsutism was assessed using the modified Ferriman–Gallwey (mFG) scoring system, with a score of ≥8 considered indicative of clinically significant hirsutism.
2. Body composition was assessed by bioelectrical impedance analysis using the Tanita MC-780 device (Tanita Corporation, Tokyo, Japan). The following parameters were evaluated: body fat percentage, fat mass (kg), fat-free mass (FFM), and muscle mass (kg).
3. Biochemical parameters: total cholesterol (TC), high-density lipoprotein cholesterol (HDL), low-density lipoprotein cholesterol (LDL), triglycerides (TG), and glucose concentrations were measured. Glucose levels were determined at fasting (GLU 0′) and 120 min after the oral glucose tolerance test (OGTT; GLU 120′).
4. Hormonal parameters: fasting (INS 0′) and 120 min post-OGTT (INS 120′) insulin concentrations were measured. The homeostasis model assessment of insulin resistance (HOMA-IR) was calculated using the formula:

$$\mathrm{HOMA-IR}=\frac{\mathrm{fasting}\;\mathrm{glucose}\;(\mathrm{mg}/\mathrm{dL})\times \mathrm{fasting}\;\mathrm{insulin}\;(\mu U/\mathrm{mL})}{22.5}.$$

The triglyceride–glucose (TyG) index, a validated indicator of insulin resistance, was calculated as:

$$\mathrm{TyG}=ln(\frac{\mathrm{fasting}\;\mathrm{triglycerides}\;(\mathrm{mg}/\mathrm{dL})\times \mathrm{fasting}\;\mathrm{glucose}\;(\mathrm{mg}/\mathrm{dL})}{2}).$$

Quantitative Insulin Sensitivity Check Index (QUICKI) was calculated using the following formula:

$$\mathrm{QUICKI}=\frac{1}{log({\mathrm{INS}}_{0}\;[\mu U/mL])+log({\mathrm{GLU}}_{0}\;[mg/dL])}.$$

The adipokine profile included adiponectin (ADPN), apelin, leptin, omentin, resistin, retinol-binding protein 4 (RBP-4), visfatin, and vaspin. The leptin-to-adiponectin ratio (L/A) was also calculated.

For the determination of biochemical and hormonal parameters, venous blood samples were collected during the follicular phase of the menstrual cycle (days 2–5) or three months after the last menstrual period, in the morning, after an overnight fast. Glucose concentrations were determined by ultraviolet photometry based on an enzymatic reaction catalysed by hexokinase (Beckman Coulter DxC 700 AU, Brea, CA, USA). TC, LDL, and HDL concentrations were measured using an enzymatic colorimetric assay (Beckman Coulter DxC 700 AU). TG concentrations were quantified using a colorimetric enzymatic assay for the determination of TG levels in human serum and plasma (Beckman Coulter DxC 700 AU). Testosterone concentration was measured using an immunochemical chemiluminescence method on the Cobas e 402 analyser (Roche Diagnostics, Mannheim, Germany).

Plasma concentrations of selected adipokines were determined using commercially available enzyme-linked immunosorbent assay (ELISA) kits according to the manufacturers’ instructions. Omentin, vaspin, adiponectin and leptin levels were measured with the Human ELISA Kit (BioVendor– Laboratorní medicína a.s., Brno, Czech Republic, no. RD191100200R, RD191097200R, RD191023100, RD191001100, respectively). Visfatin concentrations were quantified using the Human ELISA Kit (Sandwich ELISA, HRP-labelled antibody; RAG004R). Apelin concentrations were assessed using the Apelin-36 (Human) EIA Kit (extraction-free, EKE-057-15; Phoenix Pharmaceuticals). Resistin levels were measured using the Human Resistin ELISA Kit (R&D Systems, DRSN00). The concentration of retinol-binding protein (RBP4) was evaluated using the Retinol-Binding Protein ELISA Kit (Immundiagnostik, K6110).

Auxological and hormonal data were analysed using Statistica software version 13.3 PL (StatSoft, Inc., Tulsa, OK, USA). The normality of data distribution was verified using the Shapiro–Wilk test. In cases where the null hypothesis of normality could not be rejected (*p* > 0.05) in both the study and control groups, comparisons between groups were performed using the two-sample *t*-test. When the assumption of normality was violated in one or both groups (*p* < 0.05), the nonparametric Mann–Whitney U test was applied.

Descriptive statistics were computed for all variables. Data are expressed as mean ± standard deviation (SD) for normally distributed variables and as median [interquartile range (IQR = Q3-Q1)] for non-normally distributed variables. 95% confidence intervals (95% CI) were calculated. For qualitative variables, the chi-squared test (χ^2^) or Fisher’s exact test was applied where appropriate. Correlations between quantitative variables were assessed using Pearson’s correlation coefficient, with significance verified by the *t*-test. A *p*-value < 0.05 was considered statistically significant. In both groups, missing data were not imputed; the tests were conducted based on the actual sample size for each variable/feature.

## 3. Results

The clinical and biochemical characteristics of the study (SG) and control (CG) groups are presented in Table 2 and Table 3. As the SG and CG were matched for both chronological and gynecological age, no differences were observed in these parameters. However, the mean age at menarche was significantly lower in the CG compared with the SG (*p* = 0.049).

There were no significant differences in BMI z-score between the groups (*p* = 0.052); however, the SG demonstrated a significantly greater waist circumference (*p* = 0.007), a higher percentage of body fat (*p* = 0.02), and a greater absolute fat mass (*p* = 0.02) compared with the CG.

With respect to lipid metabolism, the SG exhibited significantly higher TG concentrations (*p* < 0.001) and HDL levels (*p* = 0.002) than the CG. No statistically significant differences were observed between the SG and CG in fasting or OGTT glucose and insulin levels, nor in HOMA-IR or QUICKI indices. However, the TyG index was significantly higher in the SG (*p* < 0.001).

**Figure 1:**
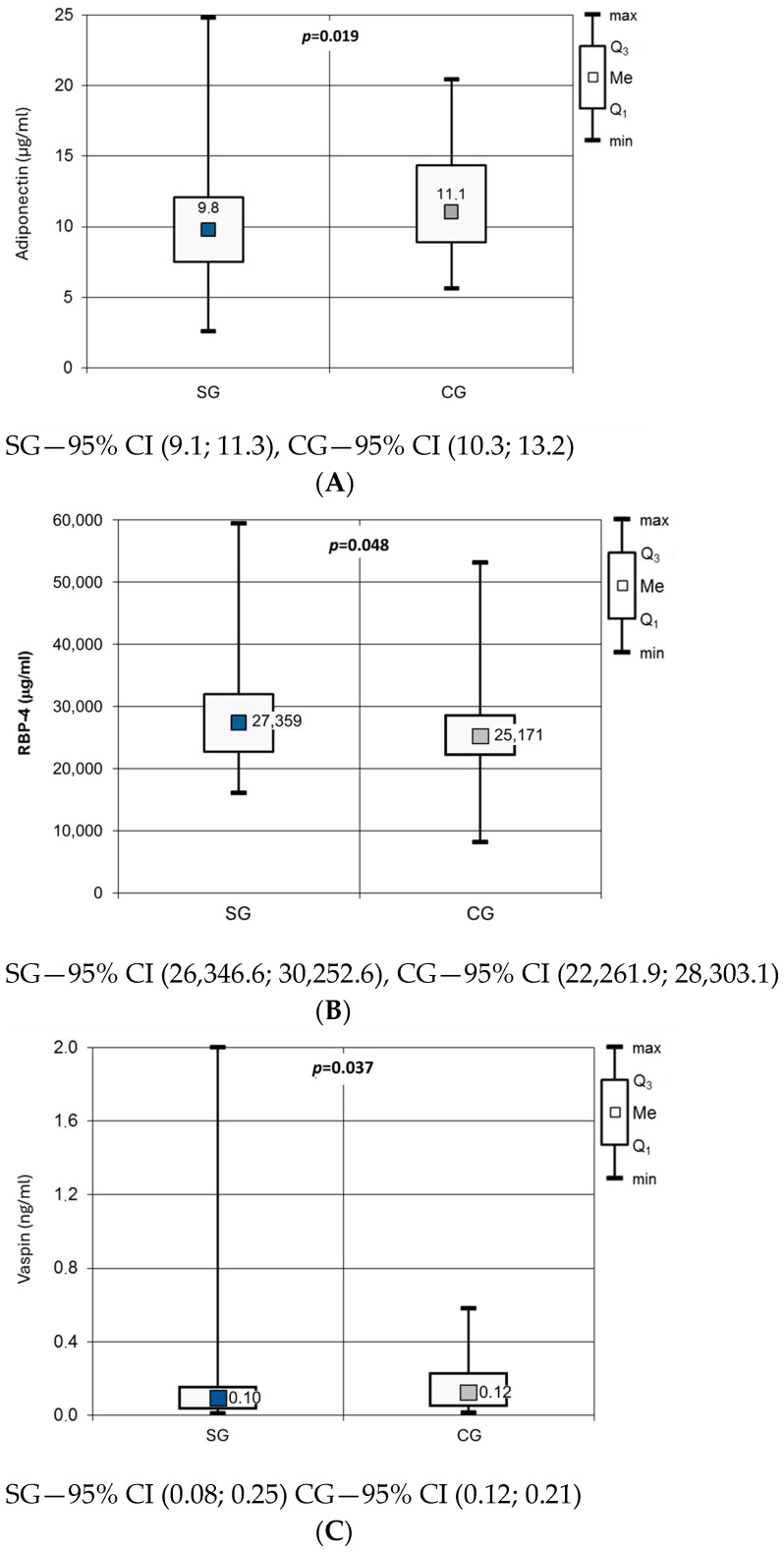
Adiponectin (**A**), RBP-4 (**B**) and vaspin (**C**) concentration in studied groups.

Evaluation of the adipokine profile in the SG compared with the CG showed significantly lower concentrations of ADPN (10.23 ± 4.54 µg/mL vs. 11.76 ± 3.88 µg/mL; *p* = 0.019), higher concentrations of RBP-4 (28,300 ± 7945 µg/mL vs. 25,283 ± 8089 µg/mL; *p* = 0.048), and lower concentrations of vaspin [Me = 0.096 (0.039; 0.154) vs. Me = 0.123 (0.058; 0.228); *p* = 0.037] (Figure 1). There were no significant differences between the groups in apelin, leptin, omentin, resistin, visfatin, or the L/A ratio (Table 4).

Correlations between adipokine concentrations and clinical and biochemical parameters in the SG are shown in Table 5, Table 6 and Table 7. Significant positive correlations were observed between anthropometric parameters [BMI z-score, WHtR, and fat mass (%)] and the concentrations of leptin, visfatin, and the L/A ratio, and negative correlations with ADPN and omentin levels (Table 4).

Very similar relationships were observed for carbohydrate metabolism (Table 6). Fasting and OGTT insulin levels, HOMA-IR, and the TyG index correlated positively with leptin and the L/A ratio, and negatively with ADPN and omentin concentrations, whereas QUICKI showed the opposite pattern. Only resistin was positively associated with fasting glucose levels.

Regarding lipid metabolism, HDL levels correlated with several adipokines (positively with ADPN, apelin, and omentin; negatively with leptin, visfatin, and the L/A ratio) (Table 7). Omentin showed a negative, and leptin and the L/A ratio a positive, relationship with triglyceride concentration, whereas resistin and visfatin levels were negatively correlated with total cholesterol, and resistin also with LDL levels. Moreover, for visfatin and omentin, the relationships within lipid metabolism were reversed in the study group (SG) compared with those observed in the control group (CG) (as shown in the Appendix A).

## 4. Discussion

Numerous studies have suggested a relationship between abnormal adipokine secretion and both the pathophysiology and prevalence of metabolic disorders in women with PCOS. The aim of the present study was to evaluate the adipokine profile in adolescent girls with PCOS and to investigate the associations between individual adipokine concentrations and parameters of carbohydrate and lipid metabolism.

Our findings demonstrated that the adipokine profile of adolescent girls with PCOS differs from that of regularly menstruating girls, particularly with respect to ADPN, RBP-4, and vaspin concentrations. Both anthropometric adiposity indices and INS levels, as well as indices of IR, correlated positively with leptin and the L/A ratio and negatively with ADPN concentrations. Furthermore, positive correlations were observed between ADPN, apelin, and omentin, while negative correlations were noted between leptin, the L/A ratio, and HDL levels. Among anthropometric, lipid, and carbohydrate metabolism parameters, the strongest correlation coefficients were found for leptin and the L/A ratio. Interestingly, only resistin concentrations correlated positively with GLU 0′ and negatively with TC and LDL levels.

Adiponectin, which plays a central role in the mechanisms underlying the development of insulin resistance, is considered a potential biomarker for assessing the risk of PCOS and its associated metabolic complications [8]. In addition to its insulin-sensitising properties, ADPN exhibits anti-inflammatory, anti-atherosclerotic, anti-apoptotic, pro-angiogenic, and pro-adipogenic effects [9]. In our study, we observed significantly lower ADPN concentrations in the SG compared with the CG, consistent with previous reports, including those conducted in adolescent populations [10,11]. Moreover, significant negative correlations between ADPN and both BMI z-score and body fat percentage confirm the association between adiposity and reduced ADPN levels in girls with PCOS.

Another adipokine of interest, RBP-4, demonstrates sexual dimorphism, with higher levels observed in males, suggesting a modulatory effect of androgens on its secretion [12,13]. Elevated serum RBP-4 concentrations have been reported in obesity, insulin resistance, and T2DM [12,13]. RBP-4 is also considered an independent predictor of cardiovascular disease in women [14]. Therefore, the association between RBP-4 and IR may represent a potential mechanistic link in the development of PCOS. In our study, RBP-4 levels were significantly higher in girls with PCOS than in controls, consistent with findings from other studies [15,16].

Vaspin, another adipokine under investigation, is involved in steroidogenesis, oocyte maturation, angiogenesis, cell proliferation, and apoptosis. As an important regulator of gonadal function, vaspin may serve as a potential marker of ovarian dysfunction, including PCOS. Increased expression of vaspin and its receptor, GRP78, has been observed in granulosa cells and follicular fluid of women with PCOS. In the present study, we found significantly lower vaspin concentrations in the SG compared with the CG, which contrasts with findings reported by other researchers [17,18]. Thus, data regarding vaspin levels in PCOS remain inconsistent and warrant further investigation.

### 4.1. Relationship of Adipokine Networks to Parameters of Carbohydrate Metabolism

Our study revealed negative correlations between ADPN and omentin concentrations and both fasting and post-oral glucose tolerance test (OGTT) insulin (INS) levels, as well as positive correlations with the QUICKI. In addition, omentin concentrations were negatively correlated with the HOMA-IR and the TyG index. These findings support the role of ADPN and omentin as endogenous enhancers of tissue sensitivity to insulin.

One of the few studies investigating adipokine levels in adolescent female patients with PCOS also demonstrated a negative association between ADPN levels and insulinaemia [19], as well as a positive correlation with glucose concentrations [20]. Mirza et al. similarly confirmed a negative relationship between insulin resistance (IR) and ADPN concentrations, suggesting that IR may represent a mechanistic link between hypoadiponectinaemia and PCOS [11].

With respect to omentin, a study in adolescent girls with PCOS reported no association between its concentrations and parameters of carbohydrate metabolism [21]. In contrast, a large cross-sectional study by Yang et al. in adult non-obese women with PCOS demonstrated inverse correlations between omentin levels and both HOMA-IR and fasting insulin (INS 0′). The presence of IR was associated with lower plasma omentin concentrations in non-obese women with PCOS compared with those without IR [22]. Tan et al. presented further compelling evidence, showing a dose-dependent, insulin- and glucose-induced increase in omentin mRNA expression, protein levels, and secretion into conditioned media from adipose tissue explants. Moreover, the induction of hyperinsulinaemia in healthy subjects significantly reduced plasma omentin concentrations [23]. These findings suggest that low omentin levels may be a secondary consequence of hyperinsulinaemia and could serve as an indicator of IR and a potential risk factor for T2DM. In contrast, in our CG, no significant associations were observed between omentin concentrations and carbohydrate metabolism parameters (Appendix A), which may suggest different regulatory mechanisms in metabolically healthy individuals.

In addition, our study demonstrated positive correlations between leptin concentrations and the L/A ratio with INS 0′ and INS 120′, HOMA-IR, and the TyG index. The L/A ratio was also negatively correlated with QUICKI. Similar associations were observed by Peng et al. [24]. Insulin is known to increase leptin mRNA expression and stimulate leptin secretion in adipocytes; thus, elevated leptin levels in women with PCOS may represent a secondary effect of hyperinsulinaemia. Conversely, the development of leptin resistance—closely linked to obesity—promotes triglyceride accumulation in adipose tissue, liver, skeletal muscle, and pancreas, leading to impaired insulin secretion, decreased insulin sensitivity, and ultimately, the development of IR.

Regarding the L/A ratio, previous studies have suggested that it may be superior to conventional indices of IR or insulin sensitivity, such as HOMA-IR, QUICKI, the fasting glucose-to-insulin ratio, or the McAuley index [25]. The L/A ratio has also been reported to correlate more strongly with IR than ADPN or leptin alone [26]. Our findings support the potential utility of the L/A ratio as a biomarker of IR and impaired glucose metabolism, even among adolescents with PCOS. However, in our study, the L/A ratio correlated with carbohydrate metabolism parameters and IR indices to a similar extent as leptin.

Hyperresistinaemia inhibits insulin signalling, enhances gluconeogenesis and glycogenolysis, and reduces glucose uptake; conversely, a reduction in resistin levels improves insulin sensitivity and glucose tolerance [27]. In our study, resistin concentrations correlated positively only with fasting glucose (GLU 0′), which further supports its antagonistic role in insulin regulation [27,28].

In summary, adipokines associated with carbohydrate metabolism parameters include ADPN, leptin, omentin, and the L/A ratio. These may serve as potential biomarkers of metabolic dysfunction and represent a link between obesity and insulin resistance in the pathogenesis of PCOS.

### 4.2. Relationship of Adipokine Networks to Lipid Metabolism Parameters

Our analysis revealed a higher prevalence of an unfavourable lipid profile in SG. Girls with PCOS were characterised by significantly elevated TG levels and reduced HDL concentrations. Among the adipokines examined, only RBP-4 and vaspin showed no significant regulatory association with lipid profile parameters.

Based on our findings, higher concentrations of ADPN, apelin, omentin, and a lower L/A ratio were associated with increased HDL concentrations, suggesting a more favourable lipid profile in the PCOS group. The effect of ADPN on lipoprotein metabolism has also been confirmed by other studies, with the antiatherogenic properties primarily attributed to its high-molecular-weight (HMW) fraction. Serum ADPN, particularly its HMW form, has been reported to correlate positively with HDL and negatively with TG concentrations [29,30]. In contrast, Altinkaya et al. [31] observed a positive correlation between apelin and TG levels and an inverse relationship between apelin and HDL concentrations in PCOS patients, while other researchers found no such associations [32]. Thus, the relationship between apelin and lipid metabolism in PCOS remains inconclusive.

In our study, serum omentin levels in the SG were positively correlated with HDL and negatively correlated with TG concentrations, suggesting a potential role of this adipokine in promoting a more favourable lipid profile in PCOS patients. However, another study in adolescent girls with PCOS reported no association between omentin levels and lipid abnormalities [21].

We also observed negative correlations between leptin and visfatin concentrations and HDL levels, as well as a positive correlation between leptin and TG concentrations. Daghestani et al. similarly demonstrated a positive correlation between leptin and TC, TG, and LDL levels, along with a negative correlation with HDL in women with PCOS [33], supporting the potential contribution of leptin to lipid dysregulation in PCOS. Furthermore, other studies have shown a positive association between visfatin and lipid parameters, including TC, LDL, TG, and lipoprotein(a) levels [34].

Although the L/A ratio is primarily recognised as a marker of insulin resistance (IR), it may also serve as a better predictor of adipose tissue dysfunction than individual adipokine concentrations. The L/A ratio has been shown to correlate negatively with BMI and to be significantly reduced in patients with metabolic syndrome [35]. Recent studies have emphasised its relevance in the context of obesity, IR, coronary artery disease, and stroke [36]. In our study, we found a negative correlation between L/A and HDL and a positive correlation between L/A and TG, indicating that higher L/A values were associated with a less favourable lipid profile. Gupta et al. similarly reported a negative correlation between L/A and HDL in women with PCOS and metabolic syndrome, as well as a positive correlation with TG levels in PCOS patients without metabolic syndrome [37]. These findings suggest that this ratio reliably reflects lipid abnormalities in PCOS.

Resistin has been implicated in the regulation of adipose tissue metabolism by promoting preadipocyte proliferation, stimulating lipolysis, and inhibiting adiponectin secretion from adipocytes [38]. In our study, resistin concentrations were negatively correlated with TC and LDL levels. However, other authors have not demonstrated similar associations between resistin and lipid parameters, such as TC, LDL, or HDL [39], leaving the role of this adipokine uncertain.

Contrary to expectations, we did not observe a significant association between RBP-4 levels and lipid metabolism parameters in girls with menstrual disorders. Nonetheless, other studies have suggested a potential role of RBP-4 in lipid metabolism, particularly in triglyceride regulation [12].

A limitation of our study is its cross-sectional design and the relatively small size of both the study and control groups. Increasing the number of participants would allow for the identification of subgroups within the SG and CG, comprising individuals with normal body weight and those who are overweight or obese. This, in turn, would enable a more detailed analysis of the relationships and regulatory mechanisms of adipokine secretion depending on weight status and the presence of PCOS. An advantage of our study is the analysis of the adipokine network as a whole rather than the assessment of individual adipokines alone. Furthermore, this is one of the few studies to evaluate the adipokine profile in adolescent patients with PCOS.

## 5. Summary and Conclusions

The present study demonstrated that the adipokine profile in adolescent girls with menstrual disorders and hyperandrogenism differs from that observed in girls with regular menstrual cycles, particularly in terms of adiponectin, RBP-4 and vaspin concentrations. It appears that ADPN, leptin, and the L/A ratio are primarily influenced by nutritional status rather than by PCOS status itself.

Concentrations of ADPN, apelin, leptin, omentin, resistin, visfatin, and the L/A ratio were found to correlate with parameters of lipid metabolism in girls diagnosed with menstrual disorders and hyperandrogenism. In contrast, adipokines associated with carbohydrate metabolism included ADPN, leptin, omentin, resistin, visfatin, and the L/A ratio. These findings suggest that these adipokines may serve as potential markers of metabolic disturbances, linking obesity and insulin resistance in the pathogenesis of PCOS.

Conversely, RBP-4 and vaspin concentrations do not appear to be reliable indicators for assessing metabolic abnormalities associated with PCOS. The observed differences in the relationships between certain adipokines (RBP-4, omentin, visfatin) and anthropometric as well as metabolic parameters may indicate altered regulation of adipokine secretion or activity in girls with menstrual disorders and hyperandrogenism compared with those exhibiting regular menstrual cycles.

These findings underscore the high degree of complexity in the interactions between adipokines and metabolic disturbances in PCOS. Discrepancies in results reported by other investigators may reflect this complexity, highlighting the need for further comprehensive research to elucidate the mechanisms underlying these associations. To better understand the relationship between adipokine profile disturbances and the pathogenesis and development of metabolic complications in adolescents with PCOS, longitudinal studies evaluating these parameters over time are warranted.

## Figures and Tables

**Table 1:** Diagnostic parameters of PCOS in the analysed groups (qualitative variables).

		Group	
		SG (*n* = 66)	CG (*n* = 30)
Type of menstrual disorder	Oligomenorrhea	39 (59.1%)	0
	Primary amenorrhea	1 (1.5%)	0
	Secondary amenorrhea	26 (39.4%)	0
Regular menstrual cycles		0	30 (100%)
Hirsutism score ≥ 8		30 (45.5%)	0
Testosterone > 42 ng/dL		57 (86.4%)	10 (33.3%)

**Table 2:** Clinical characteristics of the study groups.

	Study Group (*n* = 66)	Control Group (*n* = 30)	*p*
Chronological age (years)	Me = 16.8; IQR (15.5; 17.5) 95% CI (16.3; 16.8)	Me = 16.6; IQR (15.6; 17.1) 95% CI (15.6; 16.7)	0.129 *
Gynaecological age (years)	4.2 ± 1.6 95% CI (3.8; 4.6)	4.3 ± 1.4 95% CI (3.8; 4.8)	0.396
Age of first menstruation (years)	Me = 12.0; IQR (12.0; 13.0) 95% CI (12.0; 12.7)	11.87 ± 1.68 95% CI (11.2; 12.5)	0.049 *
BMI (kg/m^2^)	Me = 26.1; IQR (21.1; 32.7) 95% CI (25.8; 29.3)	Me = 22.9; IQR (21.3; 27.4) 95% CI (22.4; 26.1)	0.043 *
BMI z-score	Me = 1.20; IQR (0.06; 1.99) 95% CI (0.77; 1.29)	0.68 ± 0.94 95% CI (0.33; 1.03)	0.052 *
Waist circumference (m)	Me = 0.83; IQR (0.70; 0.97) 95% CI (0.80; 0.89)	Me = 0.72; IQR (0.69; 0.83) 95% CI (0.72; 0.79)	0.007 *
WHtR	Me = 0.51; IQR (0.43; 0.58) 95% CI (0.49; 0.54)	Me = 0.44; IQR (0.42; 0.50) 95% CI (0.44; 0.48)	0.011 *
Fat mass on BIA (kg)	Me = 25.4; IQR (15.2; 42.4) 95% CI (25.2; 33.9)	Me = 17.8; IQR (14.9; 26.9) 95% CI (17.6; 25.5)	0.024 *
Fat mass on BIA (%)	Me = 37.1; IQR (28.3; 44.8) 95% CI (33.8; 39.3)	Me = 29.2; IQR (26.1; 37.1) 95% CI (28.7; 34.3)	0.019 *

BMI—body mass index; WHtR—waist to height ratio; BIA—bioimpedance. *—*p* of Mann–Whitney test; Me—Median; IQR—Interquartile range; 95% CI—Confidence interval.

**Table 3:** Characteristics of lipid and carbohydrate metabolism in the study groups.

	Study Group (*n* = 66)	Control Group (*n* = 30)	*p*
Total cholesterol (mg/dL)	Me = 166.5; IQR (153; 182.3) 95% CI (159.7; 175.6)	166.0 ± 33.1 95% CI (153.1; 178.8)	0.329 *
LDL cholesterol (mg/dL)	96.7 ± 26.4 95% CI (89.8; 103.6)	94.6 ± 28.7 95% CI (83.5; 105.7)	0.366
HDL cholesterol (mg/dL)	Me = 46.8; IQR (40.7; 56.2) 95% CI (45.9; 51.5)	55.0 ± 10.2 95% CI (51.0; 59.0)	0.002 *
Triglycerides (mg/dL)	Me = 101.0; IQR (79.5; 134.8) 95% CI (104.4; 131.7)	Me = 74.5; IQR (69.8; 92.3) 95% CI (73.9; 91.0)	<0.001 *
GLU 0′ (mg/dL)	Me = 88.0; IQR (85.0; 93.0) 95% CI (87.1; 90.5)	88.5 ± 6.7 95% CI (86.0; 91.0)	0.352 *
GLU 120′ (mg/dL)	Me = 106.5; IQR (94.0; 122.5) 95% CI (104.6; 119.9)	108.2 ± 27.4 95% CI (97.3; 119.0)	0.330 *
INS 0′ (uIU/mL)	Me = 13.3; IQR (7.7; 19.3) 95% CI (12.8; 18.0)	Me = 11.1; IQR (8.3; 18.1) 95% CI (11.0; 17.3)	0.448 *
INS 120′ (uIU/mL)	Me = 67.4; IQR (48.0; 122.0) 95% CI (75.6; 110.0)	Me = 68.7; IQR (55.6; 99.4) 95% CI (66.8; 122.2)	0.384 *
HOMA-IR	Me = 2.93; IQR (1.54; 4.05) 95% CI (2.82; 4.04)	Me = 2.42; IQR (1.69; 3.82) 95% CI (2.38; 3.93)	0.438 *
QUICKI	Me = 0.33; IQR (0.31; 0.36) 95% CI (0.32; 0.34)	Me = 0.33; IQR (0.31; 0.35) 95% CI (0.32; 0.35)	0.438 *
TyG index	8.47 ± 0.44 95% CI (8.36; 8.58)	8.17 ± 0.27 95% CI (8.06; 8.27)	<0.001

GLU 0′—fasting glucose, GLU 120′—glycemia in the 120th minute of the oral glucose tolerance test, INS 0′—fasting insulin concentration, INS 120′—insulin concentration in the 120th minute of the oral glucose tolerance test, HOMA-IR—homeostatic model assessment for insulin resistance, QUICKI—quantitative insulin sensitivity check index, TyG index—triglyceride-glucose index. *—*p* of Mann–Whitney test; Me—median; IQR—Interquartile range; 95% CI—Confidence interval.

**Table 4:** Adipokine profile in the study groups.

	Study Group (*n* = 66)	Control Group (*n* = 30)	*p*
apelin (ng/mL)	Me = 0.124; IQR (0.088; 0.186) 95% CI (0.123; 0.161)	0.155 ± 0.078 95% CI (0.125; 0.184)	0.177 *
leptin (ng/mL)	Me = 38,5; IQR (17.4; 68.4) 95% CI (42.4; 63.8)	Me = 34.1; IQR (20.3; 49.6) 95% CI (29.3; 50.3)	0.161 *
omentin-1 (ng/mL)	413.3 ± 153.9 95% CI (375.4; 451.1)	Me = 401.3; IQR (335.8; 565.6) 95% CI (394.4; 565.7)	0.213 *
resistin (ng/mL)	Me = 6.62; IQR (5.66; 9.85) 95% CI (6.85; 9.70)	7.70 ± 2.82 95% CI (6.65; 8.76)	0.495 *
visfatin (ng/mL)	Me = 1.32; IQR (0.77; 2.88) 95% CI (1.63; 2.55)	Me = 1.26; IQR (0.66; 2.34) 95% CI (1.17; 2.78)	0.247 *
L/A	3.97; IQR (1.71; 9.63) 95% CI (4.90; 8.23)	Me = 3.14; IQR (1.54; 4.51) 95% CI (2.52; 5.12)	0.059 *

L/A—leptin to adiponectin ratio. *—*p* of Mann–Whitney test; Me—median; IQR—Interquartile range; 95% CI—Confidence interval.

**Table 5:** Correlations between adipokine levels and anthropometric parameters in the study group.

	BMI z-Score	Waist Circumference	WHtR	Fat Mass on BIA (%)
adiponectin	−0.253 (*p* = 0.020) 95% CI (−0.487; −0.020)	−0.274 (*p* = 0.025) 95% CI (−0.537; −0.012)	−0.331 (*p* = 0.008) 95% CI (−0.584; −0.078	−0.327 (*p* = 0.01) 95% CI (−0.584; −0.071)
leptin	0.652 (*p* < 0.001) 95% CI (0.508; 0.795)	0.736 (*p* < 0.001) 95% CI (0.606; 0.866)	0.737 (*p* < 0.001) 95% CI (0.607; 0.867)	0.804 (*p* < 0.001) 95% CI (0.702; 0.905)
omentin	−0.391 (*p* = 0.001) 95% CI (−0.603; −0.180)	−0.293 (*p* = 0.018) 95% CI (−0.552; −0.033)	−0.335 (*p* = 0.008) 95% CI (−0.587; −0.083)	−0.316 (*p* = 0.010) 95% CI (−0.575; −0.058)
visfatin	0.370 (*p* = 0.001) 95% CI (0.155; 0.586)	0.415 (*p* = 0.001) 95% CI (0.180; 0.650)	0.446 (*p* < 0.001) 95% CI (0.219; 0.674)	0.424 (*p* = 0.001) 95% CI (0.189; 0.660)
L/A	0.633 (*p* < 0.001) 95% CI (0.483; 0.783)	0.715 (*p* < 0.001) 95% CI (0.576; 0.854)	0.750 (*p* < 0.001) 95% CI (0.625; 0.874)	0.782 (*p* < 0.001) 95% CI (0.671; 0.894)

WHtR—waist to height ratio, BIA—bioimpedance, L/A—leptin to adiponectin ratio; 95% CI—Confidence interval. Observations: no significant correlations in the study group between apelin, RBP-4, resistin, vaspin levels and anthropometric parameters.

**Table 6:** Correlations between adipokine concentrations and parameters of carbohydrate metabolism (a) and insulin resistance/sensitivity indices (b) in the study group.

(a) Correlations between adipokines levels and parameters of carbohydrate metabolism in the study group							
	GLU 0′		GLU 120′		INS 0′		INS 120′
adiponectin	0.003 (*p* = 0.490) 95% CI (−0.248; 0.255)		−0.077 (*p* = 0.279) 95% CI (−0.339; 0.184)		−0.237 (*p* = 0.029) 95% CI (−0.475; 0.001)		−0.215 (*p* = 0.048) 95% CI (−0.463; 0.034)
leptin	0.092 (*p* = 0.233) 95% CI (−0.157; 0.342)		0.028 (*p* = 0.416) 95% CI (−0.235; 0.291)		0.481 (*p* < 0.001) 95% CI (0.287; 0.674)		0.332 (*p* = 0.004) 95% CI (0.100; 0.564)
omentin	−0.198 (*p* = 0.057) 95% CI (−0.440; 0.044)		−0.018 (*p* = 0.445) 95% CI (−0.281; 0.244)		−0.274 (*p* = 0.013) 95% CI (−0.507; −0.042)		−0.322 (*p* = 0.006) 95% CI (−0.556; −0.089)
resistin	0.384 (*p* < 0.001) 95% CI (0.169; 0.599)		0.041 (*p* = 0.379) 95% CI (−0.222; 0.303)		−0.011 (*p* = 0.467) 95% CI (−0.262; 0.241)		−0.068 (*p* = 0.300) 95% CI (−0.328; 0.191)
visfatin	0.114 (*p* = 0.183) 95% CI (−0.134; 0.363)		0.068 (*p* = 0.303) 95% CI (−0.194; 0.33)		0.108 (*p* = 0.196) 95% CI (−0.141; 0.357)		0.055 (*p* = 0.337) 95% CI (−0.205; 0.315)
L/A	0.040 (*p* = 0.374) 95% CI (−0.211; 0.292)		0.082 (*p* = 0.267) 95% CI (−0.179; 0.343)		0.461 (*p* < 0.001) 95% CI (0.263; 0.659)		0.435 (*p* < 0.001) 95% CI (0.224; 0.646)
(b) Correlations between adipokines levels and parameters of insulin resistance/sensitivity in the study group							
		HOMA-IR		QUICKI		TyG index	
adiponectin		−0.223 (*p* = 0.037) 95% CI (−0.463; 0.016)		0.239 (*p* = 0.028) 95% CI (0.002; 0.476)		−0.051 (*p* = 0.345) 95% CI (−0.304; 0.202)	
leptin		0.451 (*p* < 0.001) 95% CI (0.250; 0.652)		−0.493 (*p* < 0.001) 95% CI (−0.683; −0.302)		0.259 (*p* = 0.019) 95% CI (0.022; 0.496)	
omentin		−0.283 (*p* = 0.011) 95% CI (−0.515; −0.051)		0.331 (*p* = 0.004) 95% CI (0.107; 0.556)		−0.219 (*p* = 0.041) 95% CI (−0.460; 0.023)	
resistin		0.025 (*p* = 0.421) 95% CI (−0.226; 0.277)		−0.108 (*p* = 0.195) 95% CI (−0.357; 0.14)		0.080 (*p* = 0.265) 95% CI (−0.172; 0.332)	
visfatin		0.091 (*p* = 0.235) 95% CI (−0.158; 0.341)		−0.223 (*p* = 0.037) 95% CI (−0.462; 0.016)		−0.057 (*p* = 0.238) 95% CI (−0.310; 0.196)	
L/A		0.427 (*p* < 0.001) 95% CI (0.221; 0.633)		−0.473 (*p* < 0.001) 95% CI (−0.668; −0.277)		0.313 (*p* = 0.006) 95% CI (0.084; 0.542)	

L/A—leptin to adiponectin ratio, GLU 0′—fasting glucose, GLU 120′—glycemia in the 120th minute of the oral glucose tolerance test, INS 0′—fasting insulin concentration, INS 120′—insulin concentration in the 120th minute of the oral glucose tolerance test, HOMA-IR—homeostatic model assessment for insulin resistance, QUICKI—quantitative insulin sensitivity check index, TyG index—triglyceride-glucose index; 95% CI—Confidence interval. Observations: no significant correlations in the study group between apelin, RBP−4 and vaspin levels and carbohydrate metabolism parameters.

**Table 7:** Correlations between adipokines levels and lipid metabolism parameters in the study group.

	Total Cholesterol	LDL Cholesterol	HDL Cholesterol	Triglycerides
Adiponectin	−0.048 (*p* = 0.352) 95% CI (−0.302; 0.205)	−0.203 (*p* = 0.061) 95% CI (−0.458; 0.051)	0.267 (*p* = 0.017) 95% CI (0.031; 0.503)	−0.093 (*p* = 0.233) 95% CI (−0.345; 0.159)
Apelin	0.122 (*p* = 0.168) 95% CI (−0.128; 0.372)	0.020 (*p* = 0.439) 95% CI (−0.245; 0.286)	0.213 (*p* = 0.046) 95% CI (−0.030; 0.455)	0.067 (*p* = 0.299) 95% CI (−0.186; 0.320)
Leptin	0.160 (*p* = 0.104) 95% CI (−0.088; 0.407)	0.213 (*p* = 0.053) 95% CI (−0.041; 0.466)	−0.309 (*p* = 0.006) 95% CI (−0.539; −0.08)	0.225 (*p* = 0.037) 95% CI (−0.016; 0.466)
Omentin	−0.019 (*p* = 0.441) 95% CI (−0.273; 0.235)	−0.078 (*p* = 0.280) 95% CI (−0.341; 0.186)	0.270 (*p* = 0.015) 95% CI (0.035; 0.506)	−0.222 (*p* = 0.039) 95% CI (−0.464; 0.019)
Resistin	−0.341 (*p* = 0.003) 95% CI (−0.565; −0.116)	−0.296 (*p* = 0.011) 95% CI (−0.538; −0.054)	−0.200 (*p* = 0.057) 95% CI (−0.444; 0.044)	−0.022 (*p* = 0.433) 95% CI (−0.275; 0.232)
Vaspine	−0.021 (*p* = 0.436) 95% CI (−0.274; 0.233)	−0.020 (*p* = 0.440) 95% CI (−0.285; 0.245)	−0.053 (*p* = 0.338) 95% CI (−0.306; 0.200)	0.016 (*p* = 0.451) 95% CI (−0.238; 0.270)
Visfatin	−0.231 (*p* = 0.033) 95% CI (−0.472; 0.009)	−0.197 (*p* = 0.067) 95% CI (−0.452; 0.058)	−0.236 (*p* = 0.030) 95% CI (−0.476; 0.004)	−0.054 (*p* = 0.337) 95% CI (−0.307; 0.199)
L/A	0.153 (*p* = 0.113) 95% CI (−0.095; 0.401)	0.210 (*p* = 0.056) 95% CI (−0.044; 0.463)	−0.361 (*p* = 0.002) 95% CI (−0.582; −0.14)	0.300 (*p* = 0.008) 95% CI (0.069; 0.531)

L/A—leptin to adiponectin ratio; 95% CI—Confidence interval. Observations: no significant correlation in the study group between RBP-4 levels and lipid metabolism parameters.

## Data Availability

The datasets generated and analyzed during the current study are not publicly available due to privacy restrictions, but are available from the corresponding author upon reasonable request.

## Appendix A

Below are presented the correlations between the concentrations of individual adipokines in CG and the anthropometric parameters (Table A1), carbohydrate metabolism parameters (Table A2), and lipid metabolism parameters (Table A3).

**Table A1:** Correlations between adipokine levels and anthropometric parameters in the control group.

	BMI z-Score	Waist Circumference	WHtR	Fat Mass on BIA (%)
adiponectin	−0.404 (*p* = 0.013) 95% CI (−0.728; −0.080)	−0.355 (*p* = 0.027) 95% CI (−0.693; −0.016)	−0.372 (*p* = 0.022) 95% CI (−0.705; −0.038)	−0.140 (*p* = 0.242) 95% CI (−0.544; 0.263)
leptin	0.501 (*p* = 0.002) 95% CI (0.211; 0.791)	0.604 (*p* = 0) 95% CI (0.358; 0.850)	0.422 (*p* = 0.01) 95% CI (0.104; 0.740)	0.593 (*p* = 0.001) 95% CI (0.325; 0.860)
omentin	−0.225 (*p* = 0.116) 95% CI (−0.593; 0.142)	−0.124 (*p* = 0.256) 95% CI (−0.505; 0.257)	−0.131 (*p* = 0.246) 95% CI (−0.511; 0.25)	−0.198 (*p* = 0.161) 95% CI (−0.594; 0.197)
visfatin	0.254 (*p* = 0.088) 95% CI (−0.108; 0.616)	0.293 (*p* = 0.058) 95% CI (−0.061; 0.647)	0.305 (*p* = 0.051) 95% CI (−0.047; 0.656)	0.115 (*p* = 0.283) 95% CI (−0.291; 0.522)
L/A	0.509 (*p* = 0.002) 95% CI (0.222; 0.796)	0.638 (*p* < 0.001) 95% CI (0.409; 0.868)	0.444 (*p* = 0.007) 95% CI (0.133; 0.755)	0.492 (*p* = 0.005) 95% CI (0.179; 0.804)

WHtR—waist to height ratio, BIA—bioimpedance, L/A—leptin to adiponectin ratio; 95% CI—Confidence interval.

**Table A2:** Correlations between adipokine concentrations and parameters of carbohydrate metabolism (a) and insulin resistance/sensitivity indices (b) in the control group.

(a) Correlations between adipokines levels and parameters of carbohydrate metabolism in the control group							
	GLU 0′		GLU 120′		INS 0′		INS 120′
adiponectin	−0.199 (*p* = 0.146) 95% CI (−0.571; 0.173)		−0.220 (*p* = 0.135) 95% CI (−0.612; 0.172)		−0.064 (*p* = 0.371) 95% CI (−0.457; 0.329)		−0.187 (*p* = 0.176) 95% CI (−0.584; 0.211)
leptin	0.337 (*p* = 0.034) 95% CI (−0.006; 0.680)		0.465 (*p* = 0.007) 95% CI (0.142; 0.788)		0.341 (*p* = 0.035) 95% CI (−0.008; 0.690)		0.252 (*p* = 0.102) 95% CI (−0.134; 0.638)
omentin	−0.033 (*p* = 0.432) 95% CI (−0.419; 0.354)		−0.332 (*p* = 0.045) 95% CI (−0.699; 0.034)		−0.056 (*p* = 0.386) 95% CI (−0.45; 0.338)		−0.265 (*p* = 0.091) 95% CI (−0.648; 0.118)
resistin	0.165 (*p* = 0.191) 95% CI (−0.211; 0.542)		0.190 (*p* = 0.171) 95% CI (−0.207; 0.587)		0.187 (*p* = 0.166) 95% CI (−0.194; 0.568)		0.144 (*p* = 0.238) 95% CI (−0.260; 0.547)
visfatin	0.06 (*p* = 0.375) 95% CI (−0.325; 0.446)		−0.045 (*p* = 0.413) 95% CI (−0.456; 0.367)		−0.062 (*p* = 0.375) 95% CI (−0.455; 0.331)		−0.079 (*p* = 0.347) 95% CI (−0.489; 0.33)
L/A	0.417 (*p* = 0.011) 95% CI (0.098; 0.737)		0.549 (*p* = 0.002) 95% CI (0.261; 0.837)		0.451 (*p* = 0.007) 95% CI (0.137; 0.766)		0.366 (*p* = 0.030) 95% CI (0.009; 0.723)
(b) Correlations between adipokines levels and parameters of insulin resistance/sensitivity in the control group							
		HOMA-IR		QUICKI		TyG index	
adiponectin		−0.099 (*p* = 0.304) 95% CI (−0.49; 0.291)		−0.071 (*p* = 0.358) 95% CI (−0.464; 0.322)		−0.050 (*p* = 0.4) 95% CI (−0.452; 0.352)	
leptin		0.352 (*p* = 0.031) 95% CI (0.005; 0.698)		−0.333 (*p* = 0.039) 95% CI (−0.684; 0.018)		−0.11 (*p* = 0.289) 95% CI (−0.508; 0.288)	
omentin		−0.063 (*p* = 0.372) 95% CI (−0.457; 0.330)		0.028 (*p* = 0.442) 95% CI (−0.366; 0.423)		0.331 (*p* = 0.043) 95% CI (−0.028; 0.690)	
resistin		0.216 (*p* = 0.131) 95% CI (−0.161; 0.592)		−0.122 (*p* = 0.263) 95% CI (−0.511; 0.266)		−0.006 (*p* = 0.488) 95% CI (−0.409; 0.397)	
visfatin		−0.047 (*p* = 0.404) 95% CI (−0.441; 0.347)		0.271 (*p* = 0.078) 95% CI (−0.095; 0.637)		0.297 (*p* = 0.063) 95% CI (−0.071; 0.664)	
L/A		0.472 (*p* = 0.005) 95% CI (0.166; 0.779)		−0.364 (*p* = 0.026) 95% CI (−0.706; −0.021)		0.005 (*p* = 0.49) 95% CI (−0.398; 0.408)	

L/A—leptin to adiponectin ratio, GLU 0′—fasting glucose, GLU 120′—glycemia in the 120th minute of the oral glucose tolerance test, INS 0′—fasting insulin concentration, INS 120′—insulin concentration in the 120th minute of the oral glucose tolerance test, HOMA-IR—homeostatic model assessment for insulin resistance, QUICKI—quantitative insulin sensitivity check index, TyG index—triglyceride-glucose index; 95% CI—Confidence interval.

**Table A3:** Correlations between adipokines levels and lipid metabolism parameters in the control group.

	Total Cholesterol	LDL Cholesterol	HDL Cholesterol	Triglycerides
Adiponectin	−0.201 (*p* = 0.153) 95% CI (−0.587; 0.186)	−0.345 (*p* = 0.036) 95% CI (−0.7; 0.010)	0.309 (*p* = 0.055) 95% CI (−0.055; 0.674)	0.029 (*p* = 0.442) 95% CI (−0.374; 0.432)
Apelin	0.254 (*p* = 0.096) 95% CI (−0.123; 0.631)	0.309 (*p* = 0.055) 95% CI (−0.056; 0.673)	−0.112 (*p* = 0.285) 95% CI (−0.510; 0.286)	0.165 (*p* = 0.201) 95% CI (−0.227; 0.557)
Leptin	0.232 (*p* = 0.117) 95% CI (−0.149; 0.614)	0.338 (*p* = 0.039) 95% CI (−0.019; 0.695)	−0.115 (*p* = 0.280) 95% CI (−0.513; 0.283)	−0.194 (*p* = 0.161) 95% CI (−0.582; 0.194)
Omentin	−0.213 (*p* = 0.139) 95% CI (−0.597; 0.172)	−0.138 (*p* = 0.242) 95% CI (−0.533; 0.258)	−0.464 (*p* = 0.006) 95% CI (−0.78; −0.147)	0.379 (*p* = 0.023) 95% CI (0.034; 0.724)
Resistin	0.068 (*p* = 0.365) 95% CI (−0.333; 0.47)	0.057 (*p* = 0.386) 95% CI (−0.345; 0.459)	0.098 (*p* = 0.310) 95% CI (−0.301; 0.497)	−0.085 (*p* = 0.334) 95% CI (−0.485; 0.316)
Vaspine	0.163 (*p* = 0.203) 95% CI (−0.229; 0.555)	0.182 (*p* = 0.177) 95% CI (−0.208; 0.572)	−0.038 (*p* = 0.425) 95% CI (−0.440; 0.365)	0.126 (*p* = 0.261) 95% CI (−0.270; 0.523)
Visfatin	0.466 (*p* = 0.006) 95% CI (0.15; 0.781)	0.456 (*p* = 0.007) 95% CI (0.137; 0.776)	0.09 (*p* = 0.325) 95% CI (−0.31; 0.49)	0.316 (*p* = 0.051) 95% CI (−0.047; 0.679)
L/A	0.262 (*p* = 0.089) 95% CI (−0.114; 0.637)	0.420 (*p* = 0.013) 95% CI (0.088; 0.752)	−0.279 (*p* = 0.075) 95% CI (−0.651; 0.092)	−0.120 (*p* = 0.272) 95% CI (−0.517; 0.277)

L/A—leptin to adiponectin ratio; 95% CI—Confidence interval.
